# Flavine and Brilliant Green Powerful Antiseptics with Low Toxicity to the Tissues: Their Use in the Treatment of Infected Wounds

**Published:** 1917-11

**Authors:** 


***Flavine and Brilliant Green Powerful Antiseptics with Low Toxicity to the Tissues : Their Use in the Treatment of Infected Wounds.*** By C. C. Browning, R. Gulbransen, E. L. Kennaway, L. H. D. Thornton, all of the Bland-Sutton Institute of Pathology, the Middlesex Hospital. Abstracted from the *British Medical Journal*, Jan. 20, 1917.

(Note the abstract following this one for a criticism of the theories and experiments here exposed.)

The defects and deleterious action of antiseptics now commonly in use led the authors to the investigations recorded in this report. They observed the action of carbolic acid, mercury perchloride, iodine, hypochlorites (in the form of " eusol " and also Daufresne's modification of Dakin's solution), chlorine water, malachite green, brilliant green, crystal violet, ethyl violet, and flavine.

They believe that in flavine they have found a compound which more nearly attains to the ideal antiseptic than any now in use. They claim for it that by the presence of serum it is made more efficient, whereas most antiseptics lose in potency in undiluted serum. To quote: "This is the only one of *high* antiseptic potency which more than maintains its efficiency in serum." They also speak very favorably of the action of brilliant green for the same reasons, but consider flavine to be superior in most particulars.

Their conclusions are based both upon laboratory experiments and upon a considerable clinical experience. Their conclusions are supported by an article in the same number of the *B. M. J.* by D. Ligat, Acting Assistant Surgeon to the Middlesex Hospital. This author declares that 150 cases, 50 o/o of which were war wounds, were treated with brilliant green or flavine with excellent results. In many of them there was infection and considerable suppuration. These acridine compounds, however, seem equally effective in prophylaxis, especially because they do not injure healthy tissues, even when injected. This surgeon has tested these antiseptics for over a year.

B. G. K. and T. describe their laboratory experiments as follows:

"The substances to be tested, in a volume usually not exceeding .1 cc., was added to 1 cc. of the culture medium, which consisted in one series of .7 o/o of peptone water and in the other of undiluted serum, and then .1 cc. of a 1 : 20,000 dilution in saline of a 24-hour peptone water culture was added. A control was made with peptone water or serum without antiseptic; one loopful of this mixture when stroked immediately upon agar yielded twenty or more colonies of staphylococcus or B. coli. The tubes were then placed at 37° C. and were examined at the end of twenty-four to forty-eight hours in order to determine the concentration of antiseptic which killed the organisms introduced; for this purpose subcultures were made on agar and also in peptone water. The results of both methods of subculture corresponded in general; but it was sometimes found that cultures containing antiseptic, which showed no turbidity after incubation and in which, therefore, little or no multiplication of organisms had occurred, still contained living bacteria.

"Ox serum heated at 57° C. was used throughout; experiments were also performed with human serum and rabbit serum. A certain amount of variation was obtained with different specimens of serum; but the results corresponded in general with those quoted in the table. Fresh rabbit serum gave practically the same result as serum heated at 57° C.

"B. coli frequently grew very poorly in serum; this difficulty was overcome by adding. 25 cc. of 10 o/o peptone water to each 1 cc. of serum.

" In a number of experiments *the time rate of sterilization* was ascertained by making subcultures from the antiseptic mixtures at intervals of two, five, and twenty-four hours. Mercury perchloride, carbolic acid, chloramine-T, and flavine were compared in this way, and it was found that the maximum action of the first three substances was exerted within a shorter time than was the case with flavine.

" *The effect of the presence of red corpuscles in the mixture with antiseptic and serum* was tested as Emery has suggested. Thus, defibrinated rabbit's blood was employed as the medium and it was found that staphylococci were killed in defibrinated blood by flavine 1 : 40,000, whereas mercuric chloride 1 : 2000 failed to sterilize the mixture.

" Pus from an empyema, which on centrifuging yielded half its volume of sediment and in culture gave a growth of staphylococci and streptococci, was just sterilized in twenty-four hours by the addition of mercury perchloride to a concentration of 1 : 1000; carbolic acid 1 : 500; brilliant green 1 : 10,000, and flavine 1 : 2000.

" In addition, Streptococcus faecalis (enterococcus) and Streptococcus pyogenes were tested with mercury perchloride, brilliant green, and flavine; the enterococcus proved the more resistant, and with it the following results were obtained :

| | Lethal concentration in | |
|---|---|---|
| | Peptone water. | Serum. |
| Mercury perchloride. . . . . | 1 : 200,000 | 1 : 10,000 |
| Brilliant green . . . . . . . | 1 : 150,000 | 1 : 10,000 |
| Flavine. . . . . . . . . . . | 1 : 10,000 | 1 : 40,000 |

" Phagocytic Experiments. — Equal quantities of human serum, washed human " leucocyte cream ", emulsions of organisms, and appropriate dilutions of antiseptic were mixed together, and then incubated in capillary tubes for twenty minutes at 37° C. At the end of this time the mixtures were spread on slides, stained, and the number of organisms ingested by fifty leucocytes estimated in each case; the control contained normal saline in place of the antiseptic. The serum and corpuscles were on every occasion obtained from the same subject. The organism employed was a twenty-four hour agar slope culture of Staphylococcus aurens emulsified in normal saline to give an homogeneous emulsion of about two thousand million organisms per cubic centimeter; the control then gave an average of twenty-five cocci per leucocyte. The dilutions of the various antiseptics were in all cases made with normal saline. The figures given in the table were confirmed by the results of at least three experiments with each antiseptic; and a number of antiseptics were investigated simultaneously so as to avoid attaching importance to any differences in the results due merely to individual variations."

The authors' results are tabulated and they comment on them as follows: "A comparison between the bactericidal power in serum and the effect on phagocytosis shows that in the case of all the antiseptics in common use, a concentration which is sufficient to cause death of the organism is also detrimental to phagocytosis. The measurement of antiphagocytic action is obviously an important point since it enables 'general toxicity to living matter' to be estimated to some extent by test tube experiments. Thus carbolic acid both kills organisms and inhibits phagocytosis at a concentration of 1 : 250 to 1 : 500, and mercury perchloride exerts both effects at 1 : 7,000 to 1 : 10,000. On the other hand brilliant green kills cocci at 1 : 30,000 and only inhibits phagocytosis at 1 : 2,000. Finally flavine kills both cocci and B. coli at a concentration of 1 : 100,000, whereas in order to affect phagocytosis a concentration greater than 1 : 500 is required. Clearly the higher the germicidal power and the lower the point of phagocytic inhibition, the more valuable antiseptic becomes."

In clinical practice the authors have found flavine to be all that laboratory results promise. They declare that it has become a custom at the Middlesex Hospital to treat all casualty cases by washing out with a 1 : 1000 solution of flavine and they find that most of the wounds so treated heal by first intention. They admit, however, that few of these injuries are of the nature of war wounds. In all about 3000 patients have been treated—half with brilliant green, and half with flavine. These solutions, and especially flavine, are equally efficacious when there is, and when there is not, suppuration. No ill effects have ever been noted from the use of flavine, even when injected into tissues. Several cases are given in detail. References are added.

